# The long-lasting impacts of the COVID-19 pandemic on population-based cancer survival: what are the implications for data analysis?

**DOI:** 10.1038/s41416-024-02931-0

**Published:** 2024-12-14

**Authors:** Rachael Stannard, Paul C. Lambert, Georgios Lyratzopoulos, Therese M.-L. Andersson, Sam Khan, Mark J. Rutherford

**Affiliations:** 1 Biostatistics Research Group, Department of Population Health Sciences, University of Leicester, Leicester, UK; 2 Cancer Registry of Norway, Norwegian Institute of Public Health, Oslo, Norway; 3 Department of Medical Epidemiology and Biostatistics, Karolinska Institutet, Stockholm, Sweden; 4 Epidemiology of Cancer Healthcare & Outcomes (ECHO), Dept. of Behavioural Science and Health, Institute of Epidemiology & Health Care (IEHC) University College London (UCL), London, UK; 5 Leicester Cancer Research Centre, University of Leicester, Leicester, UK

**Keywords:** Epidemiology, Cancer epidemiology

## Abstract

Monitoring trends of cancer incidence, mortality and survival is vital for the planning and delivery of health services, and the evaluation of diagnostics and treatment at the population level. Furthermore, comparisons are often made between population subgroups to explore inequalities in outcomes. During the COVID-19 pandemic routine delivery of health services were severely disrupted. Resources were redeployed to COVID-19 services and patient risk of COVID-19 infection required serious consideration. Cancer screening services were paused, the availability of healthcare providers was reduced and, in some cases, patients faced difficulty in accessing optimal treatment in a timely manner. Given these major disruptions, much care should be taken when interpreting changes in cancer survival estimates during this period. The impact on cancer incidence and mortality statistics that have already been reported in some jurisdictions should drive further thought on the corresponding impact on cancer survival, and whether any differences observed are real, artificial or a combination of the two. We discuss the likely impact on key cancer metrics, the likely implications for the analysis of cancer registration data impacted by the pandemic and the implications for comparative analyses between population groups and other risk factor groups when using data spanning the pandemic period.

## Introduction

Monitoring time trends in cancer incidence, mortality and survival is vital for the planning and delivery of health services, and in the evaluation of diagnostics and treatment at the population level. Furthermore, comparisons are often made between population subgroups of interest to explore inequalities in outcome, such as between regions, sexes or socio-economic groups [1].

It is well established that during the COVID-19 pandemic routine delivery of the health service was severely disrupted in many countries worldwide. Resources were redeployed to COVID-19 services and patient risk of COVID-19 infection during healthcare delivery required serious consideration. Screening services for cancer were paused, the capacity of GPs, nurses, specialist doctors and other healthcare providers was reduced for non-covid conditions and, in some cases, patients faced difficulty in accessing optimal treatment in a timely manner [2–5]. These alterations were both a necessity and a calculated compromise to maintain a functioning health service [6]. The impacts were seen across many disease areas including cancer, cardiovascular disease, kidney disease and diabetes.

The cessation of cancer screening services is likely to result in some asymptomatic cases progressing to symptomatic cases, potentially with an associated later stage of disease. Cases that were still diagnosed through screening, but many months later when services resumed, may have also progressed beyond their hypothetical disease stage at diagnosis if the pandemic had not occurred. The delays in diagnosis may result in more progressive cancer at diagnosis, likely resulting in excess deaths due to cancer [7, 8]. Once services resumed, a severe backlog remained, and it is estimated to take several years to clear [9].

As with delayed diagnosis, delayed access to treatment could result in disease progression and hence poorer survival outcomes [10]. Clinicians and patients were required to assess the risks of COVID-19 infection when preparing treatment plans. Further to delayed treatment, patients experienced suboptimal alterations to their treatment plans and delays and alterations in the management of recurrent or end stage disease [11]. Studies have reported a decline in incidence and short-term survival, and have predicted a longer-term impact [12, 13]. It may take several years to realise the full extent of the impact.

**Fig. 1:**
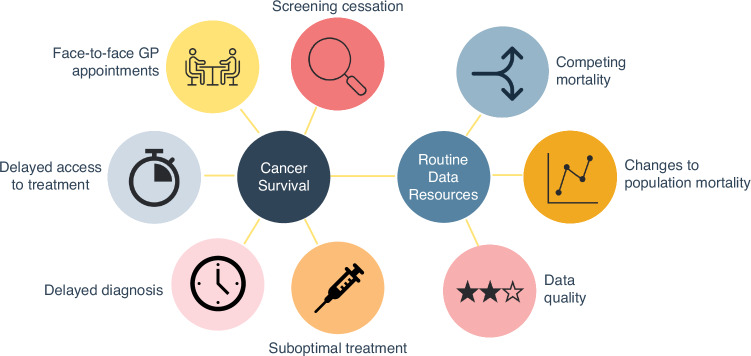
Key factors impacting the analysis of cancer registry-based data due to the COVID-19 pandemic. A visual depiction of some of the key factors to consider when assessing the impact on the data recorded in routine cancer resources and the impact on patient survival during and after the COVID-19 pandemic.

In addition to excess deaths due to cancer, there has also been an increase in population mortality rates during 2020 and 2021 compared to what would have been expected in the absence of the pandemic [14]. Estimates of background mortality rates are required for the estimation of net survival, which is often the chosen metric for population-based survival. Figure 1 illustrates some of these key factors affecting the data recorded in routine cancer resources, whilst also flagging the potential impacts on patients and their prognosis due to the pandemic.

## Impact of COVID-19 on cancer survival

It is useful to consider each group of patients at risk of cancer and undergoing active treatment. Table 1 below separates patients who at the start of the pandemic were yet to be diagnosed, both those who would usually be screen-detected and those who may be diagnosed symptomatically. The subsequent groups listed are those who were diagnosed prior to the pandemic in active treatment, and finally patients in a post-active treatment state of ‘survivorship’. For each group we consider the mechanism through which survival may be impacted, and possible approaches to study this impact.

**Table 1:** Summary of impacted mechanisms and evaluation approaches stratified by cancer patient subgroup.

Cancer patient subgroup^a^	Survival impact mechanism	Approach to study impact
Pre-diagnosis cases (Screen-detected)	Decreased detection due to disrupted screening.	Monitor routes to diagnosis. Monitor proportion of screen-detected cancer over time.
Pre-diagnosed cases (Symptomatic)	Shift towards more advanced stage due to delays in diagnosis and treatment.	Monitor stage trends over time. Consider methods to account for change in proportion of missing stage information.
Post-diagnosis cases (Active treatment with curative intent)	Modified/sub-optimal/delayed treatment. COVID-19 infection interference.	Use of and time to different curative/adjuvant treatment modalities pre-/post-/intra-pandemic.
Post-diagnosis cases (Active treatment with palliative intent)		Use of and time to different palliative treatment modalities pre-/post-/intra-pandemic.
Post-diagnosis cases (Post-active treatment survivors – ‘survivorship’)	Sub-optimal surveillance of recurrence. Modified/sub-optimal/delayed recurrence treatment. COVID-19 infection interference.	Use of and time to different curative/adjuvant/palliative treatment modalities pre-/post-/intra-pandemic. Monitor detection and treatment of recurrence.

^a^These patient subgroups can be considered as sub-cohorts of the prevalent cancer pool, including the ‘pre-prevalent’ (pre-diagnosed) cases, all such sub-cohorts impacted cross-sectionally by the pandemic as period effect.

For individuals which we describe as pre-diagnosis cases, the likely impact is more advanced disease at diagnosis due to decreased diagnostic activity. Those who would have been screen-detected in the absence of the pandemic may have become interval cases. Those who were diagnosed during the COVID-19 pandemic due to presentation of symptoms may have also received suboptimal and/or delayed treatment, further impacting survival. To study this impact, it is necessary to monitor changes to routes to diagnosis, changes in the proportion of screen-detected cancers, incidence trends by both month and year, trends of stage at diagnosis and changes to short-term survival such as 15 days or 1 month from diagnosis. We should explore methods to account for the increased proportion of missing stage information.

We separate individuals who were in active treatment, described as post-diagnosis cases, into those with curative intent or those with palliative intent. Both of these groups may have experienced suboptimal and/or delayed treatment, which can lead to poorer survival. These patients were also at a heightened risk of COVID-19 itself. COVID-19 infection interference could decrease cancer survival in two ways: a) an indirect effect, whereby the infection leads to treatment delay/modification, leading to reduced survival due to sub-optimal treatment; b) a direct effect, though a decrement in all-cause survival due to mortality from COVID-19. Separating these two possible mechanisms from the general pandemic effects relating to disruption of cancer services is challenging. Linkage of cancer registry information with COVID-19 infection population-based data may be helpful. Trends in the use of and timeliness of different treatment modalities should be studied.

The final group are those described as post-diagnosis cases who are no longer receiving active treatment. These patients may experience suboptimal recurrence surveillance, suboptimal and/or delayed recurrence treatment and COVID-19 infection interference. In addition to monitoring trends in treatment modalities, it is also necessary to monitor recurrence detection and treatment.

For each of these three patient groups, namely pre-diagnosis cases, post-diagnosis cases in active treatment and post-active cases no longer in active treatment, it is important to also study changes to these statistics by socio-economic group, age group, ethnic group and other risk factor groups.

The implications of the pandemic are wide-ranging and some may be very difficult to quantify accurately even in the fullness of time. In the remainder of this article, we consider implications for analysts planning to use any cancer registration data spanning these calendar periods, even if the focus of that study is not related in any way to COVID-19. The easiest way to frame the impact on the data collated in the cancer registration process is to contrast to the counterfactual “what if” scenario of the pandemic having never happened. We can use this thought process to hypothesise which metrics and types of analysis are most likely to be impacted.

## Implications for routine reporting and monitoring of cancer

### Data resources

Population-based cancer registry data resources, such as the National Cancer Registration and Analysis Service (NCRAS) in England [15], are a vital source of research data that are used across many research studies for epidemiological research; both in terms of monitoring trends in descriptive studies and to undertake comparative, analytical studies of risk factors and treatments. These same data resources are also increasingly used as an approach for long-term follow-up data for clinical trials [16, 17].

There will be an undoubted impact of the pandemic on these data and further the representativeness of the individuals that were added to these data resources due to the changes in clinical interactions during the pandemic. There have been reports of decreased incidence, a shift towards later stage cancer and changes in recorded patient information such as a higher proportion of unknown/not recorded cancer stage. The impact on cancer registration processes may also impact measures of cancer incidence, diagnosis, and survival at the population level.

There were also necessary changes to treatment and diagnostic practice over periods of 2020 and 2021 that will further impact on potential cancer patient survival prospects during this period and looking forwards in calendar time. Others have begun to explore the level of impact that this has had, but how to utilise these data resources in a consistent manner given the massive changes in recorded data is yet to be studied or discussed in detail. These reports of counts, descriptive statistics and estimates of short-term survival provide a critical starting point in understanding the changes to cancer incidence and mortality during the pandemic, but it is essential to also consider the impact on the estimation of long-term survival metrics, and how we fairly compare these metrics. These concerns are not limited to COVID-19 specific studies, but apply to any study using data from the period. For instance, international and risk group comparison studies utilise these electronic health databases to unpick and explain disparities, with the view to these being removed. To make fair comparisons, it is essential to account for differences due to the pandemic.

### Cancer incidence

An obvious impact for cancer incidence monitoring is the potential displacement of cases from their hypothetical incidence date should the pandemic had not have happened. This has been evidenced across several cancer sites and countries [18–22], and furthermore some evidence of a ‘catching up’ of cases has also been observed in certain settings [5, 8]. The implications for monitoring of cancer incidence in general are not particularly stark in this case – it is obvious some care must be taken to account for variation across regions and countries – but it is likely that many of these cancer cases will still have been diagnosed at some point, with the exception of those who died due to COVID-19. However, studies using the years spanning the pandemic as part of their study period do need to consider the likely case-mix of individuals that still were diagnosed with cancer during those time periods and whether that has implications for their research question of interest. It is likely that those that *would* have been detected through screening, with a potentially better stage profile overall, contribute a proportion of the ‘delayed’ cases, although there is also strong evidence that those with symptomatic disease were also not being diagnosed through GP referral pathways to the same extent in England [23].

### Cancer mortality

Even in a hypothetical scenario where cancer-specific mortality is unaffected by changes to healthcare delivery due to the COVID-19 pandemic, all-cause mortality may increase as a result of COVID-19 mortality among the population with cancer.

Cancer mortality rates among the whole population, as opposed to the population with cancer, may also have been affected. Cancer mortality rates rely on accurate recording of death certification to ascertain the underlying cause of death. For the most part, it is likely that deaths due to cancer would still be recorded consistently during this period. However, other causes of death, known as competing mortality, influence cancer mortality rates. For example, age and site-specific cancer mortality rates that decrease in 2020 may reflect the excess deaths in the population due to COVID-19, rather than a direct change in cancer mortality.

### Cancer survival

Given the real and artefactual changes to survival time discussed in this text, we must consider how to fairly compare and monitor cancer survival over time periods spanning the pandemic with metrics from pre and post-pandemic periods. In addition to the comparison of metrics over time, we must also consider how to make fair comparisons between population groups and other risk factor groups when using data spanning the pandemic period. Often our analyses concern data spanning several calendar years and hence the suitability of existing methods will require consideration for many years.

#### Delayed diagnosis

A key consideration when calculating cancer patient survival is the impact of delays in diagnosis caused by the pandemic. Care must be taken when interpreting changes in survival time during this period.

**Fig. 2:**
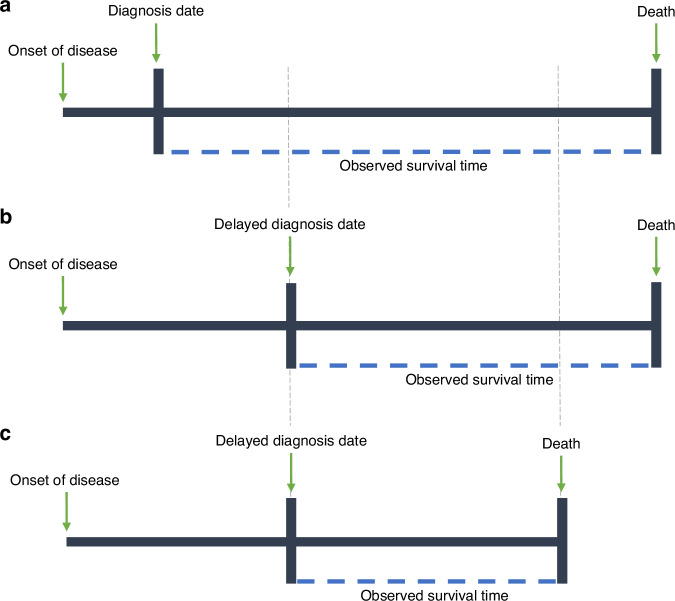
Illustration of the complexities of interpreting survival time metrics due to changes in origin. Scenario A given in (**a**) illustrates a typical timeline from onset of disease to death. Scenarios B and C, given in (**b**, **c**), illustrate two potential changes in observed survival time due to a delayed diagnosis. Scenario B illustrates a shorter observed survival time even if the delayed diagnosis does not affect the date of death. Scenario C illustrates an even greater reduction in observed survival time in the case where delayed diagnosis negatively impacts the date of death.

For example, the cessation of screening and other cancer services in many countries has resulted in diagnostic delays. Scenario A in Fig. 2 depicts what we may typically see in the absence on the pandemic. Scenarios B and C depict two potential changes in observed survival time for a delayed diagnosis. Scenario B illustrates a shorter observed survival time even if the delayed diagnosis does not affect the date of death. This is synonymous to lead time in a screening setting, but in reverse. It is well-known that lead time bias complicates the interpretation of survival-based metrics. Scenario C illustrates an even greater reduction in observed survival time in the case where delayed diagnosis negatively impacts the date of death. We would expect this to be the case if the delay in diagnosis (or access to treatment) resulted in disease progression to a more advanced stage with worse prognosis. It is necessary to consider that some of the shortening in survival time is potentially artificial (as in Scenario B), and will further worsen prognosis when comparing to unimpacted calendar periods or registries with a lesser overall impact from the pandemic.

#### Stage distribution shift

As a result of delays in diagnosis and changes to healthcare delivery the stage at diagnosis of patients diagnosed in 2020 and 2021 must also be considered to fairly compare cancer survival. There is evidence of a shift in distribution of stage at diagnosis and together with potential sub-optimal treatment plans, marginal measures of cancer survival may not be a fair representation of overall cancer survival in the population. Stage-specific survival comparisons across calendar year and between populations can provide an overview of differences in survival, and the use of stage-standardisation can provide an approach to fairly compare survival across calendar year and between populations.

A study in Wales reported not only a shift in stage distribution from early stage to late stage, but also an increase in missing stage information for some cancer sites [24]. The authors explored possible reasons for the change in completeness of the recording of stage information including decreased diagnostic and pathology activity. England has also seen an increase in missing stage information for many cancer sites in 2019 due to reduced capacity in 2020 and 2021 when the data was being processed.

#### Post-diagnosis cases during active treatment

In addition to patients diagnosed in or shortly after March 2020, we must consider those diagnosed beforehand who were still receiving treatment. It is possible that these patients also experienced competing mortality due to COVID-19, and changes to their treatment plans and healthcare interactions. A reduction in the number of patients treated by adjuvant therapy is likely to increase recurrence rates and reduce survival [25], but this may take several years or more to be detectable. Changes in mortality rates among the whole population may affect estimates of cancer survival. This is discussed in more detail in the next section.

#### Non-cancer mortality

Cancer patient survival from population-based cancer registry data is typically reported using net survival metrics, and these are usually estimated in the relative survival framework [26–28]. Relative survival is estimated using the all-cause survival of cancer patients, whilst accounting for the expected survival of the population in the absence of cancer. The expected survival is obtained from mortality rates given in population lifetables. Relative survival estimates assume these mortality rates are derived from a population which reflects the survival likely to be seen by the cohort of cancer patients in the absence of cancer [29, 30]. The COVID-19 pandemic has resulted in larger expected mortality rates and disproportionately affected cancer patients. Hence, it is important to assess the need to adjust standard lifetables, and how to best make these adjustments. Individuals that were considered vulnerable to COVID-19 were strongly advised to shield for prolonged periods for reasons such as age and morbidities such as a cancer diagnosis [31, 32]. Individuals who shielded successfully may have been at a lower risk of COVID-19 mortality, whilst others may have been at an increased risk due to immune deficiency and the need to attend healthcare appointments. Hence, it is possible that cancer patients experienced different COVID-19 mortality and therefore different non-cancer mortality than the general population. If we believe patients with cancer experienced greater non-cancer mortality than the general population and standard lifetables continued to be used, this would lead to an underestimate of relative survival. Furthermore, older patients are at an increased risk of mortality due to both COVID-19 and cancer, potentially resulting in non-negligible bias from inappropriate lifetables [33]. If we believe patients with cancer experienced lower non-cancer mortality than the general population and used standard lifetables this could lead to an overestimate of relative survival.

It is necessary to investigate the suitability of existing lifetables spanning 2020-2021 and subsequent years, with the possibility of adjusted lifetables for this period improving estimates of cancer survival. Patients diagnosed prior to the COVID-19 pandemic may also be impacted by these lifetables in the net survival setting when analysing survival beyond the years immediately following diagnosis.

Characteristics such as age and socioeconomic status may influence the case-mix of patients. COVID-19 mortality rates vary by age and socioeconomic status, therefore removing a disproportionate number of elderly and more deprived patients from the denominator pool of patients at risk of cancer diagnosis. Hence, it is possible that the pool of patients diagnosed with cancer immediately post-COVID-19 may be somewhat younger and somewhat less deprived. It is possible that some populations may not see a full ‘catching up’ of expected annual cancer cases due to this.

## Concluding remarks

This article focusses on highlighting potential issues, rather than proposing a detailed approach of how to address them. Given the multifaceted nature of the COVID-19 pandemic, it is not yet clear how to correct for such issues. Instead, we provide an initial caution against using the survival statistics in their raw form, without a nuanced consideration of the impact of the pandemic on the key inputs for survival analysis. Future work should expand on the issues highlighted, likely through detailed simulation studies, and propose approaches for disentangling the contributions of delayed diagnosis, suboptimal treatment and varying competing mortality to quantify the overall impact of the pandemic on cancer metrics, and to obtain unbiased estimates of changes in timing of cancer diagnosis and changes in cancer survival. Some of these changes may be real and others artefactual.

We have outlined some key considerations for the analysis of population-based cancer registry data spanning the calendar periods following 2020. Many of the issues that we raise are true of electronic health records in general, and will impact research studies looking at other diseases also. The impact of the COVID-19 pandemic on cancer mortality is complex, with particular concern regarding changes to the typical timescale from onset of disease to death. We caution against undertaking cancer patient survival analysis without due consideration of these time intervals.
